# Modelling West Nile Virus and Usutu Virus Pathogenicity in Human Neural Stem Cells

**DOI:** 10.3390/v12080882

**Published:** 2020-08-12

**Authors:** Silvia Riccetti, Alessandro Sinigaglia, Giovanna Desole, Norbert Nowotny, Marta Trevisan, Luisa Barzon

**Affiliations:** 1 Department of Molecular Medicine, University of Padova, 35121 Padova, Italy; silvia.riccetti@unipd.it (S.R.); alessandro.sinigaglia@unipd.it (A.S.); giovanna.desole88@gmail.com (G.D.); marta.trevisan@unipd.it (M.T.); 2 Viral Zoonoses, Emerging and Vector-Borne Infections Group, Institute of Virology, University of Veterinary Medicine, 1210 Vienna, Austria; Norbert.Nowotny@vetmeduni.ac.at; 3 Department of Basic Medical Sciences, College of Medicine, Mohammed Bin Rashid University of Medicine and Health Sciences, Dubai Healthcare City, P.O. Box 505055, Dubai, UAE

**Keywords:** West Nile virus, Usutu virus, Zika virus, neural stem cells, human induced pluripotent stem cells, innate antiviral response, flavivirus, virus replication, inflammasome, apoptosis

## Abstract

West Nile virus (WNV) and Usutu virus (USUV) are genetically related neurotropic mosquito-borne flaviviruses, which frequently co-circulate in nature. Despite USUV seeming to be less pathogenic for humans than WNV, the clinical manifestations induced by these two viruses often overlap and may evolve to produce severe neurological complications. The aim of this study was to investigate the effects of WNV and USUV infection on human induced pluripotent stem cell-derived neural stem cells (hNSCs), as a model of the neural progenitor cells in the developing fetal brain and in adult brain. Zika virus (ZIKV), a flavivirus with known tropism for NSCs, was used as the positive control. Infection of hNSCs and viral production, effects on cell viability, apoptosis, and innate antiviral responses were compared among viruses. WNV displayed the highest replication efficiency and cytopathic effects in hNSCs, followed by USUV and then ZIKV. In these cells, both WNV and USUV induced the overexpression of innate antiviral response genes at significantly higher levels than ZIKV. Expression of interferon type I, interleukin-1β and caspase-3 was significantly more elevated in WNV- than USUV-infected hNSCs, in agreement with the higher neuropathogenicity of WNV and the ability to inhibit the interferon response pathway.

## 1. Introduction

West Nile virus (WNV) and Usutu virus (USUV) are phylogenetically related members of the *Flavivirus* genus, family *Flaviviridae*. The enzootic transmission cycles and geographical distribution of USUV and WNV often overlap, as both viruses are mainly transmitted by *Culex* spp. mosquitoes among a variety of migratory and resident bird species, while humans are incidental dead-end hosts [1]. First isolated in Uganda in 1937, WNV is now widespread and endemic in central and southern Europe and in North America, where it causes every year outbreaks of neuroinvasive disease in humans and horses [1]. Isolated for the first time in Africa in 1959 [2], the first records of the presence of USUV in Europe date back to 1996, when an epidemics among common blackbirds occurred in the Tuscany region of Italy [3], and to 2001, when the virus caused death among several bird species in Austria [4]. In the recent years, USUV has spread in several countries in central and western Europe that are endemic also for WNV, and its activity has increased significantly, raising concern about possible risks for human health [5,6].

While WNV is a well-recognized human pathogen, associated with a high risk of neuroinvasive disease in elderly and immunocompromised patients, USUV appears to be more pathogenic and lethal for some bird species, while it rarely causes disease in humans. Most USUV infections in humans are asymptomatic [7,8,9,10,11,12], while only a few cases of USUV neuroinvasive infections have been reported so far, characterized by the occurrence of meningoencephalitis, encephalitis or polyneuritis [13,14,15,16,17,18]. Experimental infection in mouse models confirmed the lower pathogenicity of USUV in comparison with WNV. Systemic infection in outbred laboratory NMRI mice by intraperitoneal inoculation of 10^4^ fifty-percent tissue culture infective dose (TCID50) of USUV strain Vienna 2001-blackbird induced encephalitis in 1-week-old suckling animals, but not in animals aged 2 weeks or older [19]. Histopathological analysis of the brain showed widespread neuronal death, mainly by apoptosis, and viral replication in damaged neurons [19]. Likewise, mild clinical signs were observed in outbred laboratory Swiss mice, 8 weeks old, after intraperitoneal injection with either 10^2^ or 10^4^ plaque forming units (pfu) of USUV SAAR strain, whereas suckling mice succumbed to infection in a dose dependent manner [20]. At variance, mortality was significantly higher in both sucking and adult mice similarly infected with a WNV NY-99 strain [20]. Further evidence of the low pathogenicity of USUV was provided by a study investigating a USUV DNA-based vaccine [21]. In this study, 6 week-old mice deficient of the interferon type I receptor (*ifnar^-/-^* 129SvEv mice) were infected intraperitoneally (ip) with 10^4^ pfu of USUV SAAR strain to model lethal USUV infection [21]. At variance, wild type 129SvEv mice infected with USUV neither showed signs of USUV infection nor died [21,22], with the exception of a single animal showing neurological symptoms associated with neural death in the brain and spinal cord following infection with a recent USUV isolate from Belgium [22]. However, virulence of USUV could vary among different strains [23], such as in the case of WNV, for which motifs associated with neuroinvasion have been identified [24,25,26]. In *ifnar^-/-^* mice, ip injection of USUV Africa 2 strain resulted in systemic infection and viral replication mainly in the brain and spinal cord, associated with massive infiltration of inflammatory cells, motor impairment and death [27]. 

In vitro studies showed that USUV could infect a variety of human cell lines [28] and primary murine and human neural cells, including neurons, astrocytes, microglia, and induced pluripotent stem cell (hiPSC)-derived neural stem cells (NSCs) [29] and hiPSC-derived retinal pigment epithelium [27]. In particular, USUV was shown to efficiently infect primary human astrocytes in vitro, leading to a decrease in their proliferation and induction of a strong innate antiviral response, while it led to massive apoptosis of NSCs [29]. The magnitude of USUV replication and neural cell damage was significantly higher than those of Zika virus (ZIKV) [29], a neurotropic flavivirus associated with fetal microcephaly, whose tropism for human NSCs has been extensively characterized [30,31]. In infected cells, USUV was shown to induce type I and type III interferon (IFN) production, while pretreatment with IFN type I and type III inhibited USUV replication [32]. Experiments in dendritic cells showed that USUV induced a stronger antiviral response and was more sensitive to type I and III IFNs than WNV lineage 1 and 2 [32]. In an in vitro model of the blood–brain barrier, USUV could replicate in endothelial cell and induce the release of pro-inflammatory cytokines, without altering endothelial cell integrity and barrier function [27].

In this study, we compared USUV and WNV infection in hiPSC-derived NSCs, which represent the neural progenitor cells in the developing fetal brain and in the adult brain. ZIKV was included as control in the experiments, because of its known tropism for human NSCs [30,31]. The NSCs within the hippocampus and the lateral ventricle in the adult brain provide a source of new neurons for neuron turnover and are important targets for viral infection. In our previous study, we developed in vitro models of flavivirus infection based on hiPSC-derived NSCs and neurons and demonstrated that WNV could efficiently infect both human NSCs and neurons [30]. In the present study, we showed that USUV could infect and replicate in human NSCs, but with a significantly lower efficiency and cytopathic effect than WNV. Accordingly, both USUV and WNV triggered the expression of genes involved in the innate antiviral response and inflammation, but WNV induced higher levels of IFNs type I, interleukin-1ß and caspase-3 expression.

## 2. Materials and Methods

### 2.1. Cells and Culture Protocols

#### 2.1.1. Reprogramming of Erythroblasts into hiPSCs

Human NSCs were obtained by the neural differentiation of hiPSCs, which were generated by reprogramming erythroblasts from healthy donors using Sendai virus-based vectors carrying the four Yamanaka reprogramming factors (Oct3/4, Sox2, Klf4 and c-Myc), as reported [30,33]. Blood samples were collected from the donors after they gave their informed consent. The research involving human samples was conducted in accordance with the principle of the Declaration of Helsinki and with the Review Board and Ethics Committee of Padova University Hospital. Peripheral blood mononuclear cells were separated from other blood components using Ficoll-Paque Plus (Merck Millipore, Darmstadt, Germany) and then cells were cultured in Expansion Medium to generate a large population of erythroblasts to be transduced. Expansion Medium was composed of Iscove Modified Eagle Medium (EuroClone, Milan, Italy) supplemented with L-Ascorbic Acid (Sigma-Aldrich, Merck Millipore, Darmstadt, Germany; 50 µg/mL), Stem Cell Factor (ISOkine, ORF Genetics, Kopavogur, Iceland; 50 ng/mL), interleukin-3 (R&D Systems, Minneapolis, MN, USA, 10 ng/mL), erythropoietin (R&D Systems; 2U/mL), human recombinant insulin-like growth factor-1 (R&D Systems; 40 ng/mL) and dexamethasone (Sigma-Aldrich, Merck Millipore; 1 µM). A total of 2 × 10^5^ cells were transduced with the four viral vectors at a multiplicity of infection (MOI) of 10. After addition of viral vectors, cells were plated in 6-well plates with Expansion Medium and spin-inoculated at 2250 rpm for 90 min at room temperature (RT), and then incubated for 2 h at 37 °C. After incubation, cells were centrifuged at 1500 rpm for 10 min at RT to eliminate the excess viral vectors, suspended into Expansion Medium and plated on 6-well plates coated with CF1 Mouse embryonic fibroblasts (MEFs, Thermo Fisher Scientific, Waltham, MA, USA). The plates were centrifuged at 500 rpm for 30 min at RT in order to enhance cells adhesion and finally incubated at 37 °C, 5% CO_2_. Two days after transduction, Expansion Medium was substituted with fetal-bovine serum (FBS)–induced pluripotent stem cell (iPS) medium, which is composed of Dulbecco’s Modified Eagle Medium (DMEM)/F-12 with GlutaMAX supplement, filtered fetal bovine serum 20%, non-essential amino acids (NEAA) 1%, GlutaMax 100× 1%, penicillin/streptomycin 1% (all from Thermo Fisher Scientific, Waltham, MA, USA), β-mercaptoethanol 0.1% (Sigma-Aldrich, Merck Millipore), basic fibroblast growth factor (b-FGF) 10 ng/mL (ORF Genetics), L-ascorbic acid 50 µg/mL (Sigma-Aldrich, Merck Millipore), with the addition of growth factors included in the expansion medium. On the fourth day post transduction (p.t.), growth factors were withdrawn and, at the sixth day p.t., the medium was switched with 1:1 FBS–iPS and iPS media (DMEM/F-12, GlutaMAX supplement; KnockOut Serum Replacement 20% (Thermo Fisher Scientific); non-essential amino acids (NEAA) 1%; penicillin/streptomycin 1%; β-mercaptoethanol 0.1%; b-FGF (ISOkine, ORF Genetics), which was replaced daily at a concentration of 10 ng/mL. At the eighth day p.t., cells were fed only with iPS medium with 10 ng/mL of b-FGF and relaced daily. When colonies started to emerge (at about 15–20 days p.t.), they were picked with a pipette, plated into MEF-coated 6-well plates with iPS medium with the addiction of b-FGF at 10 μM final concentration and Rho Kinase (ROCK) inhibitor Y27632 (StemMACS™ Y27632, Miltenyi Biotec) at 10 μM final concentration. iPSCs were expanded and cultured on Matrigel matrix (Corning Inc, Corning, NY, USA) in StemMACS iPS-Brew XF medium (Miltenyi Biotec). Dispase II enzyme (Thermo Fisher Scientific) was used for routine passages of hiPSCs growing in clumps.

#### 2.1.2. Differentiation of hiPSCs into NSCs

When hiPSC growing in clumps on Matrigel substrate reached a confluence of 15–25%, the medium was aspirated to remove the non-attached cells and 2.5 mL of pre-warmed complete PSC Neural Induction Medium containing Neurobasal medium and Neural Induction Supplement 50× (all from Thermo Fisher Scientific) was added to each well of the 6-well plates. The neural induction medium was changed every other day from day 0 to day 7 of neural induction. On day 7, NSCs (P0) were ready to be harvested and expanded. The PSC Neural Induction Medium was removed from the well and after washing with 1 mL of Dulbecco’s phosphate buffer saline (DPBS, Thermo Fisher Scientific), cells were detached in single cells using a StemPro Accutase enzyme (Thermo Fisher Scientific). The enzyme was diluted with DPBS in each well and the cells were collected and centrifuged. After washing with DPBS, the supernatant was discarded and the NSCs were suspended in pre-warmed Neural Expansion Medium containing 50% Neurobasal medium, 50% Advanced DMEM/F12 and Neural Induction Supplement 50× (all from Thermo Fisher Scientific) and then plated in wells previously coated with Geltrex matrix (Thermo Fisher Scientific). ROCK inhibitor was added to the cell suspension to a final concentration of 5 μM and after overnight incubation, the medium was changed to complete Neural Expansion Medium to eliminate the ROCK inhibitor. Thereafter, the Neural Expansion Medium was exchanged every other day without the ROCK inhibitor. Usually, NSCs reached confluence on days 4–6 after plating. When NSCs reached confluence, they were further expanded in complete Neural Expansion Medium, cryopreserved or differentiated into specific neural cell types. The overnight treatment with the ROCK inhibitor Y27632 at a final concentration of 5 μM was required to avoid apoptosis until passage 4.

### 2.2. Viral Strains

WNV strain AUT/2008 (GenBank KF179640), lineage 2, goshawk isolate from Austria, 2008; WNV strain ITA09 (GenBank GU011992.2), lineage 1, human isolate from Italy, 2009; USUV strain Vienna 2001 (GenBank AY453411), Europe 1 lineage, Eurasian Blackbird isolate (isolate 939/01) from Vienna; ZIKV strain H/PF/2013 (GenBank KJ776791), Asian lineage, clinical isolate from French Polynesia, 2013. Viruses were grown in Vero cells to generate viral stocks at titer of 1.7 × 10^7^ pfu/mL (WNV AUT/2008), 2.0 × 10^7^ pfu/mL (WNV ITA09), 3.6 × 10^6^ pfu/mL (USUV) and 0.7 × 10^6^ pfu/mL (ZIKV). All viruses were used at passages below 10.

### 2.3. Infections with WNV, USUV and ZIKV

NSCs were seeded on Geltrex in serum-free Neural Expansion Medium and, when they reached a confluence of 30%, cell growth medium was removed and replaced with infection medium, containing the virus diluted in DMEM with 1% penicillin/streptomycin at the specified MOI. After incubation at 37 °C and 5% CO_2_ for 1 h and 30 min, the infection medium was removed, and the cells were washed with growth medium and then maintained at 37 °C and 5% CO_2_. A lysate from uninfected Vero cells was used as a mock control in all infection experiments.

### 2.4. Analysis of Virus Replication Kinetics

Viral replication kinetics were measured in NSC culture supernatants and cell lysates collected at different time points post-infection by using quantitative real-time RT-PCR (qRT-PCR) with primers and TaqMan-probe sets specific for WNV NS5, USUV NS5 and ZIKV NS5. Viral titer was measured in cell supernatant by plaque count assay and TCID50 assay [34]. 

#### 2.4.1. Quantitative Real-Time RT-PCR Analysis of Virus RNA Load

The supernatants of NSCs infected with ZIKV, WNV and USUV were collected every day until day 7 p.i. and inactivated by MagNA Pure 96 lysis buffer (Roche Applied Sciences, Basel, Switzerland). Total nucleic acids were purified from 200 µL supernatants by using an automated Roche MagNA Pure 96 System (Roche Applied Sciences). Viral genome sequences were amplified by one-step RT-PCR on a 7900HT Fast Real-Time PCR System instrument (Thermo Fisher Scientific) using primers and TaqMan-probe sets specific for USUV NS5 [35], WNV NS5 [36], and ZIKV NS5 [37], as reported [16,38,39]. All experiments were performed at least in duplicate and three negative controls containing water were added to verify that there were no contaminations at each run. The sensitivity of the real-time PCR assay was estimated to be approximately 5 copies of genome per reaction. Viral titer was estimated by using a standard curve generated from titrated viral stocks and reported as genome copies/mL. 

#### 2.4.2. Virus Titration by 50% Tissue Culture Infective Dose (TCID50) Assay

Supernatants of NSCs infected with USUV, WNV and ZIKV at different MOIs were collected at different time points p.i. Scalar 10-fold dilutions in DMEM with antibiotics were prepared up to the 10^–8^ dilution and inoculated on Vero cells seeded in a 96 well tissue culture plate (1.5 × 10^4^ cells/well) in triplicate. Ten wells were infected with each dilution of viral stock. After an incubation of 1 h 30 min at 37 °C in 5% CO_2_, DMEM with 6% FBS and 1% penicillin/streptomycin was added to each well. When the presence of cytopathic effect (CPE) was evident, usually after 4–5 days depending on the virus, crystal violet fixing/staining solution (Sigma-Aldrich, Merck Millipore) was added to each well and incubated at RT for 30 min. The plate was washed two times in tap water by immersion in a large beaker, and then air dried at RT. TCID50 was assessed by presence or absence of the deep purple color in each well. The viral titer was calculated by the Spearman–Kärber algorithm.

T = 10 ^1+d(S-0.5)+1^ TCID50/mL

where d represents Log10 of the dilution and S is the sum of wells with CPE for a specific dilution factor.

### 2.5. End-Point RT-PCR Analysis of Differentiation Marker Expression 

NSCs were harvested by treatment with Accutase and total RNA was purified by using an RNeasy Mini Kit (Qiagen, Hilden, Germany). Upon reverse transcription, cDNA samples were used as a template to amplify by RT-PCR the neural stem cell marker genes (*NESTIN, PAX6, SOX1, SOX2*), the pluripotency marker gene as a negative control (*OCT4*) and the housekeeping gene (*ACTIN*) to normalize the data (Table 1).

### 2.6. qRT-PCR Analysis of mRNA Levels of Genes Involved in Innate Antiviral Response 

Expression of genes encoding key members of the antiviral innate immunity (i.e., IFIT1, IFIT2, MDA5, RIG-I, TLR2, TLR3, TLR7, TLR8, MAVS, IL1B, CASP1, CASP3, NF-kβ, TNFα, C-GAS, IRF3, IRF7, VIPERIN/RSAD2, IFNA1, IFNB1, IFNL1, IFNL2, IFNL3) was analyzed by qRT-PCR analysis by using either TaqMan™ probes- or SYBR^®^ green-based methods (Thermo Fisher Scientific) by using the oligonucleotide primers and probes reported in Table 2. Nucleic acids were purified from cells by using an RNeasy Mini Kit (Qiagen, Hilden, Germany). qRT-PCR was run on the 7900HT Fast Real-Time PCR System instrument (Thermo Fisher Scientific). qRT-PCR results were normalized to the housekeeping *GAPDH* (glyceraldehyde 3-phosphate dehydrogenase) mRNA and analyzed using ΔΔCt method. All assays were performed in triplicate and repeated in three independent experiments.

### 2.7. Immunofluorescence Assays

Cells were fixed in 4% paraformaldehyde solution (PFA; Sigma-Aldrich, St. Louis, MO, USA) in phosphate-buffered saline (Thermo Fisher Scientific), permeabilized with PBS/0.1% Triton X-100 (Sigma-Aldrich) and blocked in 4% bovine serum albumin (BSA, Sigma-Aldrich) in PBS. Then, cells were incubated with the primary antibodies diluted in PBS with 4% BSA. Primary antibodies specific for Flavivirus envelope protein E (clone 4G2, mouse, 1:500, Merck Millipore, USA), PAX6 (rabbit, 1:100, Sigma-Aldrich), Nestin (mouse, 1:100, Abcam, Cambridge, UK) were used. Cells were incubated overnight at 4 °C. The secondary antibodies used included the anti-mouse IgG Alexa Fluor-488 (goat, 1:250, Thermo Fisher Scientific) and the anti-rabbit IgG Alexa Fluor-546 (goat, 1:250, Thermo Fisher Scientific). DRAQ5 fluorescent probe solution (Thermo Fisher Scientific) was used to stain nuclei. Immunofluorescence was visualized by a confocal microscope (Leica, Wetzlar, Germany) under 63× magnification. 

### 2.8. Apoptosis Assay

The activity of Caspase-3 was measured at 96h p.i. in mock and infected NSCs plated in quadruplicate in 12 wells plates (7 × 10^4^/well). For each virus, an MOI of 1 was employed. Briefly, cells were detached from the well, fixed with 4% PFA (Sigma-Aldrich, USA) in PBS (Thermo Fisher Scientific), permeabilised in 90% methanol and stored at −20 °C overnight. The next day, cells were incubated at room temperature with anti-cleaved Caspase-3 (Asp175, D3E9) a primary antibody conjugated to AlexaFluor^®^ 647 fluorescent dye (rabbit mAb, Cell Signaling Technology, Danvers, MA, USA), diluted 1:100 in incubation buffer (0.5% BSA in 1× PBS). After 1 h, the antibody was removed and the cells were suspended in PBS for data acquisition with a Becton Dickinson LSR II Flow Cytometer (BD Bioscience, Franklin Lakes, NJ, USA) and analysis using Flowing software.

### 2.9. Cell Viability Assay

Cell survival was evaluated by tetrazolium dye 3-(4,5-dimethylthiazol-2-yl)-2,5-diphenyltetrazolium bromide (MTT) assay in mock and infected NSCs plated in 96 well tissue culture plate (8 × 10^3^ cells/well). Upon viral infection with a MOI of 0.1 and 1, cell viability was analyzed at day 4 and day 7 p.i. Freshly dissolved solution of MTT (5 mg/mL, AppliChem, Darmstadt, Germany) in PBS was added to each well and incubated for 4 h at 37 °C. Then, a solubilization solution (10% sodium dodecyl sulfate) and 0.01 M HCl) was added and, after an overnight incubation at 37 °C, absorbance was read at 620 nm to assess the production of formazan.

### 2.10. Statistical Analysis

Data were presented as mean value ± standard deviation. Statistical analysis was conducted using unpaired Student’s *t*-test and statistical significance was defined as *p* < 0.05. All statistical analyses were performed by using the Statistica™ software, version 13.4.0.14 (TIBCO Software Inc., Palo Alto, CA, USA). 

## 3. Results

### 3.1. Human iPSC-Derived NSCs Are Permissive to WNV and USUV Infection

**Figure 1:**
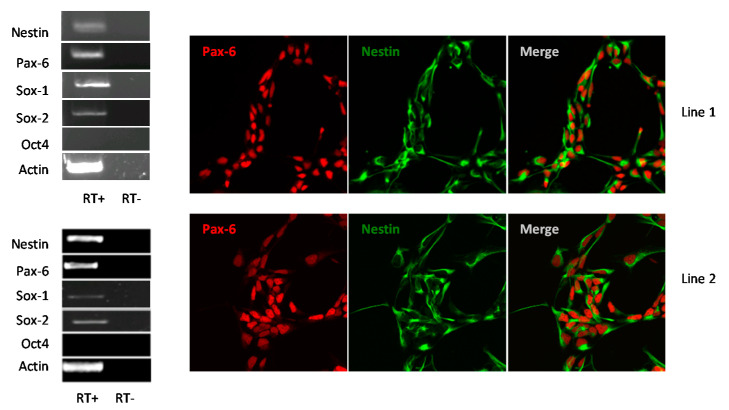
Characterization of induced pluripotent stem cell (hiPSC)s-derived neural stem cells (NSCs) lines 1 and 2: RT-PCR analysis of NSCs markers *PAX6, NESTIN, SOX1, SOX2,* the negative control *OCT4* and the housekeeping gene *ACTIN*; confocal imaging of NSCs immunolabeled for the neural progenitor markers Pax6 and Nestin. NSC Lines 1 and 2 are shown at 60× magnification. RT: Reverse transcriptase.

Human NSCs were obtained by neural differentiation of hiPSCs generated from peripheral blood mononuclear cells of two healthy donors (line 1 and line 2, respectively). The derived NSCs were characterized by detection of expression of NSC markers by RT-PCR analysis and immunofluorescence staining (Figure 1). Both NSC lines homogeneously expressed the transcription factor Pax-6 that indicates the proper neural, olfactory and ocular development, the intermediate filament Nestin that is downregulated and replaced by tissue-specific filaments in differentiated cells, and the transcription factors Sox-1 and Sox-2 that are essential for maintaining self-renewal of NSCs.

**Figure 2:**
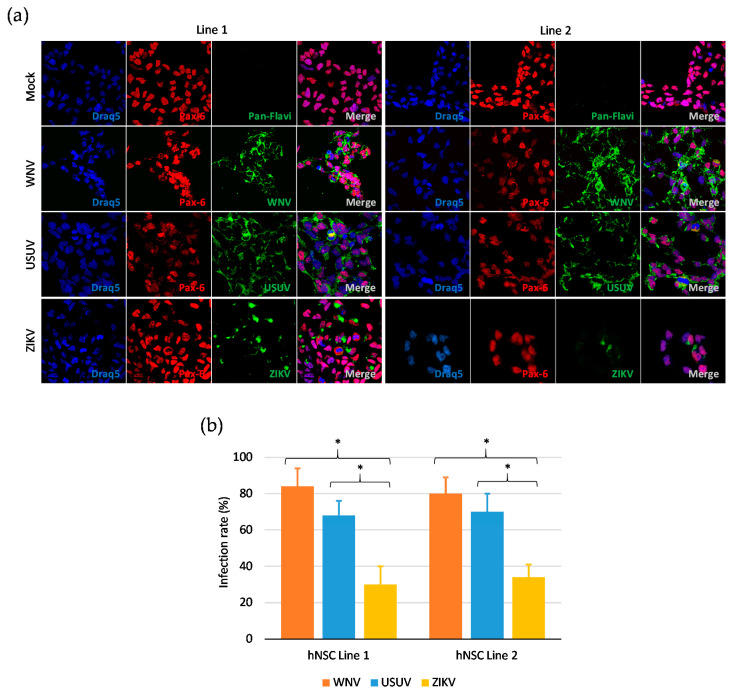
Flavivirus infection of human NSCs. (**a**) Confocal microscopy of NSCs of line 1 and line 2 at 48 hpi with West Nile virus (WNV) lineage 2, Usutu virus (USUV) Europe 1 and Zika virus (ZIKV) Asian lineage at multiplicity of infection (MOI) 1. NSCs were immunolabeled with a pan-flavivirus antibody targeting flavivirus E glycoprotein and with an antibody targeting the NSC marker Pax-6. Nuclei were stained with DRAQ5. 60× magnification zoomed two times. (**b**) Mean ± SD of the percentage of infected NSCs of line 1 and line 2 at 48 hpi with WNV, USUV and ZIKV at MOI 1. The percentage of infected cells was estimated by counting the number of immunolabeled cells to the total number of cells in 5 fields of view containing at least 100 cells, in triplicate experiments. * *p* < 0.05, Student’s *t*-test.

To determine the infection rate of flaviviruses in hNSCs *in vitro*, cells were infected with WNV, USUV and ZIKV at MOI 1 and, after 48 h, immunolabeled with a monoclonal antibody targeting the flavivirus envelope (E) glycoprotein. The percentage of infected cells was estimated by fluorescence microscopy at 60× magnification. Positive anti-E immunolabeling was detected in about 80% and 60% of hNSCs infected with WNV and USUV, respectively, thus significantly exceeding the percentage of cells infected with ZIKV, which was around 30% (Figure 2). Representative images of infected cells immunolabeled with the pan-flavivirus anti-E antibody obtained by confocal microscopy are shown in Figure 2. 

### 3.2. Kinetics of WNV, USUV, and ZIKV Production in hNSCs

**Figure 3:**
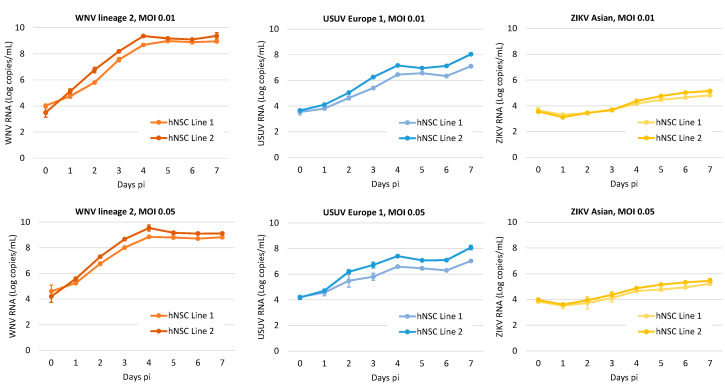
WNV, USUV and ZIKV production kinetics on hiPSC-derived NSCs. Cells were infected with WNV lineage 2, USUV Europe 1 and ZIKV Asian lineage at MOI 0.01, and 0.05 and viral RNA load was measured in cell culture supernatant by qRT-PCR daily, from day 0 to day 7 post infection (pi). Cell growth medium was replaced daily. Viral RNA load is reported as Log copies/mL. Analyses were performed in duplicate and repeated in three independent experiments. Data represent mean values ± SD of all experiments.

In this part of the study, we investigated the kinetics of virus production in the two NSC lines. Viral RNA load was measured by qRT-PCR in supernatants of NSCs infected with WNV, USUV and ZIKV at low MOIs (0.01 and 0.05) in time-course experiments (Figure 3). Since cell grown medium was replaced daily, data represent daily viral production. The kinetics of viral growth were similar in the two NSC lines. WNV replicated more efficiently than USUV and ZIKV and rapidly reached a plateau with high viral yield. ZIKV production kinetics were the least efficient among the three flaviviruses. 

### 3.3. Effects of WNV, USUV and ZIKV Infection on hNSC Viability and Caspase-3 Activity in Infected Cells

**Figure 4:**
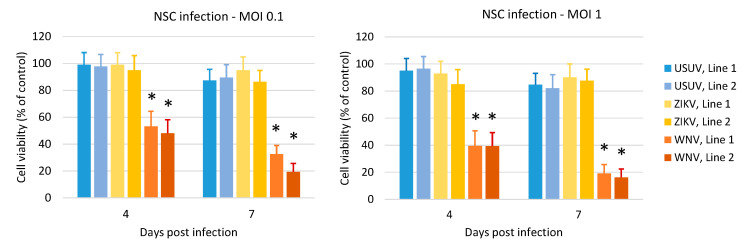
Effect of WNV, USUV and ZIKV infection on NSC viability. Cell viability was measured by MTT assay at 4 and 7 days post infection with WNV lineage 2, USUV Europe 1 and ZIKV Asian lineage at MOI 0.1 and MOI 1. Analysis was performed in eight replicates and repeated in three independent experiments. Data represent mean values ± SD of all experiments. WNV vs. USUV and vs. ZIKV, * *p* < 0.05, Student’s *t*-test.

**Figure 5:**
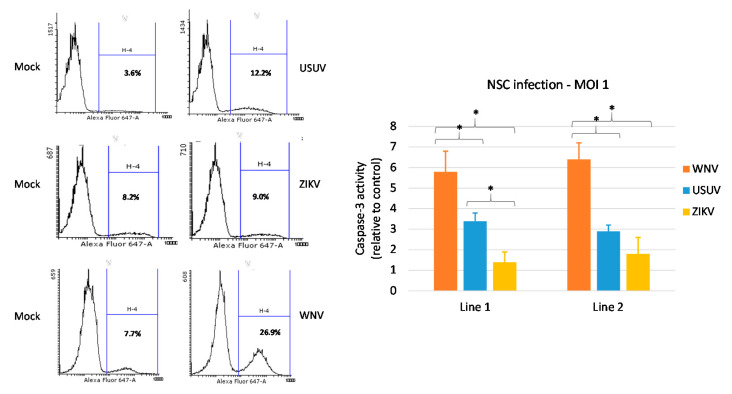
Effect of WNV, USUV and ZIKV infection on NSC apoptosis. Activated-caspase-3 was measured by flow cytometry in cells collected at 4 days post infection with WNV lineage 2, USUV Europe 1 and ZIKV Asian lineage at MOI 1. Flow cytometry data show representative experiments (infected cells are shown in the right panel and the respective mock infection control in the left panel). The experiments with the three viruses were performed independently at different times and a mock infection control was included in each experiment. Mean ± SD of caspase activity at 4 dpi of duplicate samples in triplicate experiments is reported in the graph. * *p* < 0.05, Student’s *t*-test.

Analysis of cell viability by MTT assay showed that ZIKV and USUV did not exert a significant cytopathic effect on hNSCs at 4 and 7 dpi, at variance with WNV, which strongly reduced the viability of infected cells (Figure 4). Accordingly, a stronger increase in activated-caspase 3 was observed, by flow cytometry analysis, in NSCs upon WNV infection (Figure 5). Thus, cell mortality induced by WNV infection was partly mediated by apoptotic pathway. A lower activity of caspase-3 was observed in NSCs infected with USUV and ZIKV, in agreement with the mild cytopathic effects.

### 3.4. Expression of Innate Antiviral Immune Genes in hNSCs in Response to WNV, USUV and ZIKV Infection 

**Figure 6:**
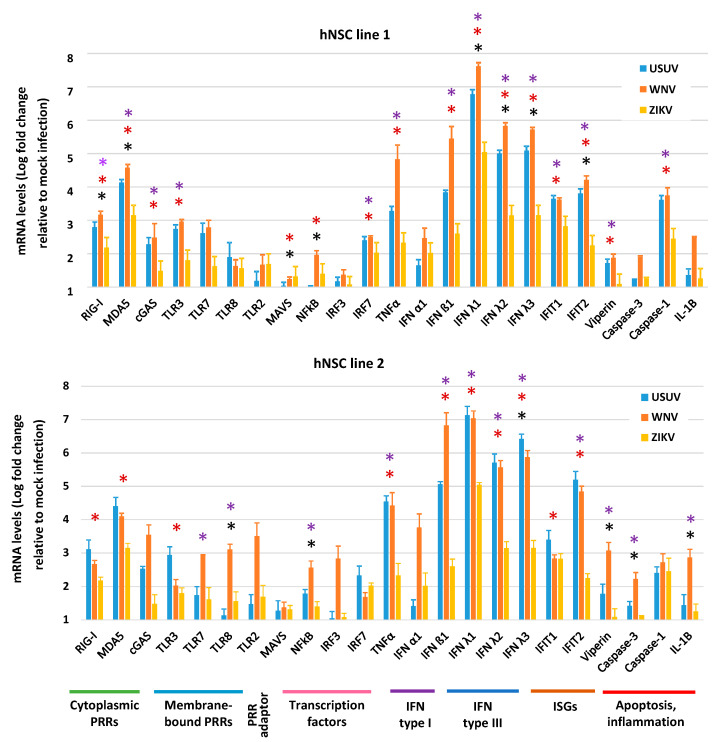
Antiviral innate immune response to WNV, USUV and ZIKV infection. qRT-PCR analysis of innate antiviral immune response gene expression in hNSC lines 1 and 2 at 4 dpi with WNV lineage 2, USUV Europe 1 and ZIKV Asian MOI 1. mRNA levels are represented as geometric mean ± SD of Log fold regulation (compared to mock control). Analyses were performed in triplicates and repeated in three independent experiments. * USUV vs. WNV; * USUV vs. ZIKV; * WNV vs. ZIKV; *p* < 0.05; Student’s *t*-test.

Host recognition of viral invasion and development of an effective antiviral innate immune response represent the first lines of defense against viral infection. However, abnormal activation of antiviral immune response and inflammation may also contribute to neurotoxicity. To characterize NSC response to viral infection, we analyzed the expression of a panel of genes, including gene encoding cellular pattern recognition receptors (PRRs) that sense RNA viruses, interferon (IFN) and IFN-stimulated genes (ISGs), and genes involved in inflammation and apoptosis. Gene expression was evaluated by qRT-PCR in hNSCs at 4 dpi with WNV, USUV and ZIKV, MOI 1. Values of mRNA Log fold regulation compared to mock infection control are shown in Figure 6. Infection with the three flaviviruses led to a general upregulation of antiviral innate immunity genes, with a particularly strong induction of IFN-λs. In both NSC lines, WNV led to the highest induction of innate immune response gene transcripts, followed by USUV and then by ZIKV. Both WNV and USUV induced high mRNA levels of the PRR genes *RIG1* and *MDA5*; the pro-inflammatory cytokine gene *TNFA*; the interferon genes *IFNB1*, *IFNL1*, *IFNL2*, and *IFNL3* (encoding for IFN-β1, IFN-λ1, IFN-λ2 and IFN-λ3, respectively); and the ISGs *IFIT1* and *IFIT2* (Figure 6). Some differences in mRNA levels were observed between WNV- and USUV-infected hNSCs, such as higher levels of *IFNB1*, *CASP3* and *IL1B* mRNAs in WNV infected cells than in USUV infected cells. At variance, in both NSC lines, WNV and USUV consistently induced significantly higher levels of *MDA5*, *TNFA*, *IFNB*, *IFNL1*, *IFNL2*, *IFNL3* and *IFIT2* mRNAs than ZIKV. 

**Figure 7:**
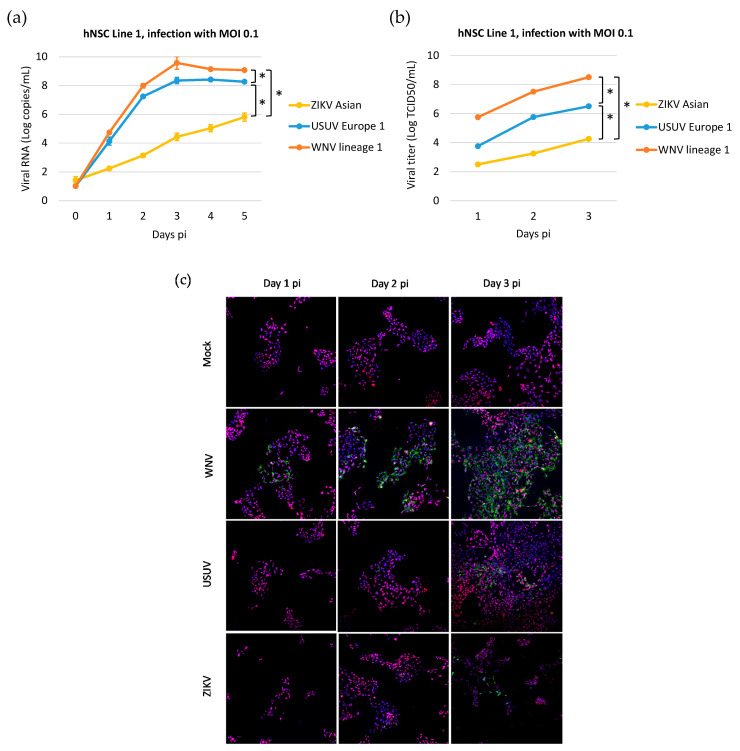
WNV, USUV and ZIKV production kinetics on hiPSC-derived human neural stem cell (hNSC) line 1. (**a**) Cells were infected with WNV lineage 1, USUV Europe 1 and ZIKV Asian at MOI 0.1 and viral RNA load was measured in cell culture supernatant by qRT-PCR daily, from day 0 to day 5 p.i.; results are reported as Log viral RNA copies/mL. Analyses were performed in triplicate and repeated in two independent experiments. (**b**) Viral titer in supernatant of NSCs infected with viruses at MOI 0.1 was measured by TCID50 assay on Vero cells on days 1, 2 and 3 p.i.; results are reported as Log TCID50/mL. Analyses were performed in triplicate and repeated in two independent experiments. Data represent mean values ± SD of all experiments. * WNV vs. USUV and vs. ZIKV, *p* < 0.05 by Student’s *t*-test. (**c**) Representative confocal microscopy images of hNSC line 1 at different days p.i. with WNV lineage 1, USUV Europe 1 and ZIKV Asian lineage at MOI 0.1 or mock infection. Cells were immunolabeled with a pan-flavivirus antibody targeting flavivirus E glycoprotein (green) and with an antibody targeting the NSC marker Pax-6 (red); nuclei were stained with DRAQ5 fluorescent probe solution (blue). Merged images are shown at 20× magnification.

**Figure 8:**
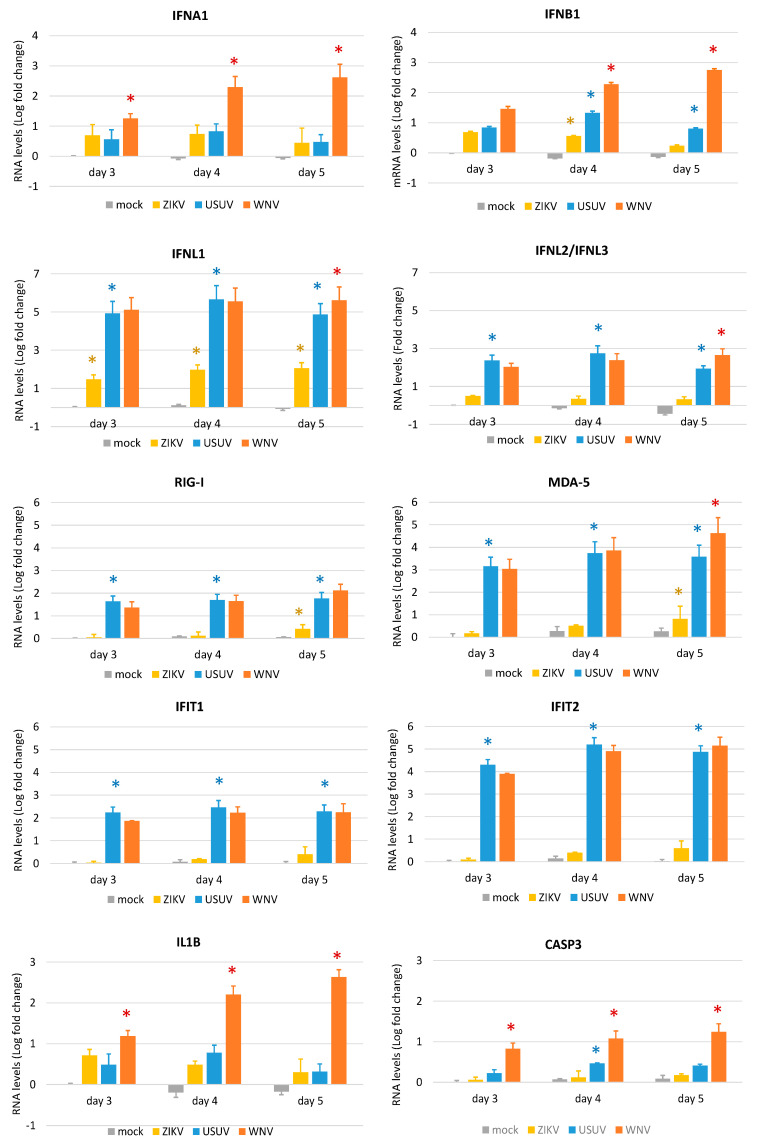
Antiviral innate immune response to WNV, USUV and ZIKV infection. qRT-PCR analysis of innate antiviral immune response gene expression in hNSC line 1 at 3, 4 and 5 dpi with WNV lineage 1, USUV Europe 1 and ZIKV Asian lineage at MOI 0.1. RNA levels are represented as geometric mean ± SD of Log fold change (compared to mock infection at day 3). Analyses were performed in quadruplicated experiments. * WNV vs. USUV; * USUV vs. ZIKV; * ZIKV vs. mock; *p* < 0.05; Student’s *t*-test.

To better characterize and compare the dynamics of WNV, USUV, and ZIKV infection and the expression of innate antiviral genes in NSCs, we performed a time course experiment of infection with a lower MOI of 0.1 in NSC line 1. In this experiment, WNV lineage 1 ITA09 was used to confirm the results obtained with WNV lineage 2 AUT/2008 in the previous experiments. Analysis of the kinetics of virus production by qRT-PCR (Figure 7a), infectious virus titration (Figure 7b), and immunolabeling of viral E glycoprotein expression (Figure 7c) showed that WNV could infect and replicate in NSCs with significantly higher efficiency than USUV and both WNV and USUV could replicate more efficiently than ZIKV. In particular, the production of infectious particles obtained from supernatants of WNV-infected NSCs was approximately 2 Logs and 4 Logs higher than in USUV- and ZIKV-infected NSCs, respectively (Figure 7b). qRT-PCR analysis of expression of IFN genes and a subset of PPRs and ISGs confirmed that both WNV and USUV induced significantly higher levels of antiviral response genes than ZIKV in infected cells, in agreement with their higher replication efficiency (Figure 8). Notably, WNV induced significantly higher levels of IFN type I (*IFNA1* and *IFNB1*), *IL1B* and *CASP3* mRNA than USUV in hNSCs, but no significant differences in the mRNA levels of the PPRs *RIG-I* and *MDA5*, the IGSs *IFIT1* and *IFIT2* and IFN-λs (Figure 8). These findings are in agreement with the higher cytopathic effects of WNV than USUV and the inhibitory activity of WNV on the IFN response pathway.

## 4. Discussion

This study evaluated the effect of WNV infection on human NSCs in comparison with USUV, a genetically-related flavivirus, which has been rarely associated with neuroinvasive disease in humans. We showed that USUV could infect NSCs, but it replicated with significantly lower efficiency and had less cytopathic effect than WNV. Both WNV and USUV triggered the overexpression of genes involved in the innate antiviral response and inflammation at similar levels, with some relevant differences (e.g., IFN type I, IL-1β, and caspase-3), which could account for the higher neuropathogenicity of WNV. In comparison, ZIKV could infect and replicate less efficiently than WNV and USUV in NSCs and induced less cytopathic effects. 

These results are in agreement with a previous study [29] reporting that iPSC-derived hNSCs are highly permissive to USUV infection, which leads to a reduction of cell viability of about 80% at day 4 p.i. and induction of caspase-3-dependent apoptosis at higher rate than after ZIKV infection. The lower susceptibility of NSCs to USUV infection and damage observed in our study could be related to differences in experimental conditions (e.g., lower MOI and lower confluence of cells at the time of infection in our study) or in the genetic background of the hiPSCs that have been used, while both studies used the same USUV 939/01 Europe 1 strain, at low passages, and propagated in Vero cells. Differences among cell clones in their susceptibility to viral infection and replication, conceivably due to polymorphisms in gene encoding factors required for viral infection and replication or involved in innate antiviral immune response [60] could be at the basis of some discrepancies of the results that can be observed between the two cell lines used in our study. Thus, these findings need to be confirmed in larger number of human NSC lines. 

USUV infected and replicated in hiPSC-derived NSCs less efficiently than WNV but induced a strong upregulation of genes involved in the innate antiviral response. It is thus conceivable that USUV infection in human NSCs, at variance with WNV, was efficiently restricted by host innate antiviral response. This hypothesis warrants further investigation in proper models, e.g., neural cells in which factors involved innate antiviral immunity have been knocked-out. Inhibition of USUV replication by type I and type III IFN has been demonstrated in vitro in human dendritic cells [30] and, indirectly, by the IFN-deficient mouse model, which is susceptible to USUV disseminated infection and neuroinvasion [20,27].

The patterns of antiviral inflammatory responses we observed in response to WNV infection and USUV infection are in agreement with data in the literature [27,29,61,62]. Both USUV and WNV induced high mRNA levels of genes involved in antiviral innate immune response and inflammation in infected hNSCs. However, some differences were observed between the two viruses, such as higher levels in WNV-infected NSCs than in USUV-infected cells of transcripts encoding type I and type III IFNs, which selectively restrict WNV neuroinvasion and pathogenesis by enhancing the integrity of the blood–brain barrier [63]. Other factors with significantly higher levels in WNV-infected cells than in USUV-infected NSCs were the apoptosis factor caspase-3, and the pro-inflammatory cytokine IL-1β, which play an important role in neural damage by flaviviruses and infection control by the host [64,65]. At variance, in both NSC lines, both WNV and USUV consistently induced significantly higher levels of several transcripts of interferon and other innate antiviral genes than ZIKV. This result suggests that USUV might be less efficient than WNV in inhibiting the antiviral response in human cells. Pathogenic flaviviruses, like WNV, DENV and ZIKV, have evolved multiple mechanisms to escape the IFN response and innate antiviral immunity [66]. Evasion strategies, which include sequestration or modification of viral RNA and inhibition of PRR signaling and downstream pathways, are mediated by flavivirus nonstructural proteins, such as NS1, NS3, NS5, and NS4B [67]. Mutations in WNV, DENV and ZIKV NS proteins associated with increased virulence and ability to suppress IFN response have been identified [67,68,69,70]. Thus, USUV variants characterized by enhanced pathogenicity could potentially emerge and this risk has increased in the recent years due to the rapid expansion of USUV circulation and activity. 

In conclusion, this study in an in vitro model of flavivirus infection on hiPSC-derived NSCs showed that USUV replicated less efficiently and induced less inflammatory response and cell damage than WNV, probably because of the higher susceptibility to host innate antiviral responses.

## Figures and Tables

**Table 1:** Oligonucleotide primers for RT-PCR analysis of cell differentiation markers.

	Target Gene	Forward/Reverse Primers (5′–3′)	References
Neural stem cells markers	*NESTIN*	GAAGGTGAAGGGCAAATCTG CCTCTTCTTCCCATATTTCCTG	[40]
	*PAX6*	TCTAATCGAAGGGCCAAATG TGTGAGGGCTGTGTCTGTTC	[41]
	*SOX1*	GCGGAAAGCGTTTTCTTTG TAATCTGACTTCTCCTCCC	[42]
	*SOX2*	TTGTCGGAGACGGAGAAGCG TGACCACCGAACCCATGGAG	[42]
Pluripotency marker	*OCT4*	GTGGAGGAAGCTGACAACAA CAGGTTTTCTTTCCCTAGCT	[43]
Housekeeping gene	*ACTIN*	GGACTTCGAGCAAGAGATGG AGCACTGTGTTGGCGTACAG	[44]

**Table 2:** Primers and probes used for qRT-PCR analysis mRNA levels of genes involved in innate antiviral response.

Target Gene	Forward/Reverse Primers/TaqMan Probe (5′–3′)	Reference
*RIG1*	ACCAGAGCACTTGTGGACGCT TGCCGGGAGGGTCATTCCTGT	[45]
*MDA5*	CAGAAGGAAGTGTCAGCTGCTTAG TGCTGCCACATTCTCTTCATCT	[46]
*CGAS*	CCTGCTGTAACACTTCTTAT TTAGTCGTAGTTGCTTCCT	[47]
*TLR3*	GAAAGGCTAGCAGTCATCCA CATCGGGTACCTGAGTCAAC	[48]
*TLR7*	CTTGGCACCTCTCATGCTCT GTCTGTGCAGTCCACGATCA	[49]
*TLR8*	AGTTTCTCTTCTCGGCCACC GGAACATGTTTTCCATGTTTCTGT	[49]
*TLR2*	GGCCAGCAAATTACCTGTGTG AGGCGGACATCCTGAACCT FAM-CCATCCCATGTGCGTGG-MGB	[50]
*MAVS*	AGCAAGAGACCAGGATCGACTG CGCAATGAAGTACTCCACCCA	[46]
*NFKB*	GCCAACAGATGGCCCATACC TGCTGGTCCCACATAGTTGC	[51]
*IRF3*	AGCAGAGGACCGGAGCAA AGAGGTGTCTGGCTGGGAAA FAM-ACCCTCACGACCCACATAAAATCTACGAGTTTG-TAMRA	[52]
*IRF7*	TACCATCTACCTGGGCTTCG AGGGTTCCAGCTTCACCA	[46]
*TNFA*	CCAGACCAAGGTCAACCTCC CCCTCCCAGATAGATGGGCT	[53]
*IFNA1*	AGAATCTCTCCTTTCTCCTG TCTGACAACCTCCCAGGCAC	[54]
*IFNB1*	GAGCTACAACTTGCTTGGATTCC CAAGCCTCCCATTCAATTGC FAM-ACAAAGAAGCAGCAATTTTCAGTGTCAGAAGCT-TAMRA	[52]
*IFNL1*	GAGGCCCCCAAAAAGGAGTC AGGTTCCCATCGGCCACATA	[49]
*IFNL2*	AATTGTGTTGCCAGTGGGGA GCGACTGGGTGGCAATAAAT	[49]
*IFNL3*	AGGGCCAAAGATGCCTTAG CAGCTCAGCCTCCAAAGC	[49]
*IFIT1*	TCTCAGAGGAGCCTGGCTAA TGACATCTCAATTGCTCCAG	[49]
*IFIT2*	AAGAGTGCAGCTGCCTGAA GGCATTTTAGTTGCCGTAGG	[49]
*RSAD2*	CTTTGTGCTGCCCCTTGAG TCCATACCAGCTTCCTTAAGCAA	[55]
*CASP1*	AAGACCCGAGCTTTGATTGACTC AAATCTCTGCCGACTTTTGTTTCC	[56]
*CASP3*	TGCATACTCCACAGCACCTG TTCTGTTGCCACCTTTCGGT	[57]
*IL1B*	GAGCAACAAGTGGTGTTCTCC AACACGCAGGACAGGTACAG	[58]
*GAPDH*	GAAGGTGAAGGTCGGAGTC GAAGATGGTGATGGGATTTC FAM-CAAGCTTCCCGTTCTCAGCC-TAMRA	[59]
